# Relationship between the *TERT, TNIP1* and *OBFC1* genetic polymorphisms and susceptibility to colorectal cancer in Chinese Han population

**DOI:** 10.18632/oncotarget.18378

**Published:** 2017-06-06

**Authors:** Chuang Li, Zixuan Zhao, Jun Zhou, Ying Liu, Hao Wang, Xinhan Zhao

**Affiliations:** ^1^ Department of Medical Oncology, The First Affiliated Hospital of Xi’an Jiaotong University, Xi’an, Shaanxi 710061, China; ^2^ Department of Interventional Therapy, Affiliated Zhongshan Hospital of Dalian University, Dalian, Liaoning 116001, China; ^3^ Elite Property Management Ltd, Saskatoon, SK S7H 0S6, Canada

**Keywords:** colorectal cancer, polymorphism, TERT, TNIP1, OBFC1

## Abstract

Colorectal cancer (CRC) is one of the most common diseases worldwide, and telomere length has been reported correlate with CRC. This study aimed to investigate whether polymorphisms of telomere length related genes are associated with susceptibility to CRC in Chinese Han population. 11 SNPs from *TERT*, *TNIP1* and *OBFC1* genes were selected and genotyped, in addition odds ratio (OR) and 95% confidence intervals (CI) were used to evaluate association between the SNPs and CRC risk in 247 patients clinically and 300 controls in a Chinese Han population. Our results showed that minor allele “G” of rs7708392 and minor allele “C” of rs10036748 in *TNIP1* gene were significantly associated with an increased the CRC risk in genotype model, dominant model and additive model after Bonferroni's multiple adjusted (*P*<0.0011). Moreover, the two SNPs rs7708392 and rs10036748 were in strong linkage disequilibrium. We observed that the haplotype “G-C” was more frequent among CRC patients and associated with a 1.58-fold increased CRC risk (95%CI=1.17-2.13, *P*=0.003). Contrarily, haplotype “C-T” was associated with a 0.63-fold reduced CRC risk (95%CI=0.47-0.86, *P*=0.003). Additionally, SNPs in this study except rs7708392 and rs10036748 were found a modest connection with CRC risk. In conclusion, our study firstly provides evidence for a novel association between polymorphisms of telomere length related *TNIP1* gene and CRC susceptibility in Chinese Han population, and the results need a further identification in a large sample size and other populations.

## INTRODUCTION

Colorectal cancer (CRC) is the third most common type of cancer and also the fourth common cause of cancer death worldwide [1]. In the United States, 160, 000 cases of CRC are diagnosed, and 57, 000 patients die per year because of CRC, making it the second leading cause of death from cancer [2]. CRC incidence and mortality undoubtedly increase the national economic burden. Thus, it is essential to uncover the molecular basis of individual susceptibility to CRC, and to confirm the initiate factors of tumor development, progression and responsiveness.

The etiology of CRC results from interactions of many factors, which were not fully understood. Study have revealed that progression accumulation of genetic and epigenetic alterations can lead to the malignant transformation of normal colonic epithelium, and genomic stability seems to be an crucial molecular in the early stage of cancer [3]. Approximately 85% of CRC are defined as chromosomal instability which is mainly driven by the telomere dysfunction [4]. Telomere is a structure that can protect chromosomes from end-to-end fusion and DNA degradation. Telomere is maintained by telomerase, a ribonucleoprotein complex containing an internal RNA component and a catalytic protein which encoded by *TERT* gene with reverse transcriptase activity. Moreover, evidence has reported that the telomere length have the strongest association with many cancer, such as bladder, esophageal, gastric and renal cancers in a meta-analysis [5]. *TERT*, *TNIP1* and *OBFC1* genes have been reported associated with telomere length in genome wide association studies (GWAS) [6, 7].

Furthermore, a variety of studies have also suggested that the polymorphisms of telomere length related genes associated with the development of cancer. For example, variants of rs10069690 and rs2242652 in *TERT* has been identified increase the risk of breast cancer [8], and another research reported that polymorphism of rs2242652 have an association with the risk of melanoma in Caucasians [9]. More and more clinical researches have focused on the association between single nucleotide polymorphism (SNP) and the disease risk, and heredity variant has been confirmed to be the primarily factor in the progression of CRC. Formerly, some articles had revealed that the mutations in the genes *NRAS*, *PI3K*, *BRAF*, *CASC8*, *SMAD7*, *DNMT1* and *XRCC3* decreases or increases risk of colorectal cancer [10–12]. In addition, study found telomere length of non-tumor tissues had a significant longer than the telomere length of CRC tissues [13]. However, only a few studies have focused on the polymorphisms of telomere length-related genes on susceptibility to CRC in the Han Chinese population. Thus, here we performed a case-control study to investigate the association between polymorphisms of the 11 SNPs in *TERT*, *TNIP1* and *OBFC1* and the risk of CRC in Chinese Han population.

## RESULTS

### Characteristic of the study participants CRC

The demographic characteristic of the study population, including gender and age are summarized in Table 1. The study included 247 CRC cases (140 females, 107 males) and 300 controls (120 females, 180 males). The ages of cases and controls were 58.32±12.75 years and 60.42±5.14 years, respectively. After statistical analysis, there were significant differences between these groups in gender (*P*<0.001) and age (*P*=0.015). Therefore, unconditional logistic regression analysis adjustment for gender and age was used to our subsequent data.

**Table 1:** Characteristic of the control individuals and patients with colorectal cancer

Characteristic	Case (N=247)	Control (N=300)	*P*-value
Gender (%)			<0.001^a^
Female	140 (56.7%)	120 (40.0%)	
Male	107 (43.3%)	180 (60.0%)	
Mean age ± SD	58.32 ± 12.75	60.42± 5.14	0.015^b^

*P*^a^ was calculated by Pearson's chi-square test; *P*^b^ was calculated by Welch's t test; *P*<0.05 indicates significant difference.

### Genotype model analysis

The basic information related to 11 SNPs in our study such as chromosomal position, allele, minor frequency (MAF), and HWE test results, appear in Table 2. We assumed that minor allele of each SNPs was a risk allele compared to wild type allele. All the SNPs were in HWE in the control groups (*P*>0.05). We then compared the differences in frequency distributions of alleles between cases and controls using Pearson Chi-Square test except rs9420907, whose frequency distributions was calculated by Continuity Correction test. The result showed that the risk allele frequency of “G” of rs7708392 was higher in cases, and it was associated with a 1.518-flod increased the CRC risk at a 5% level (OR=1.52, 95%CI=1.15-2, *P*=0.003). And, the risk allele “C” of rs10036748 also increased the risk of CRC (OR=1.52, 95%CI=1.15-2, *P*=0.003). Risk allele frequencies of the other SNPs were not had a significant difference between cases and controls.

**Table 2:** Allele frequencies of candidate SNPs among the cases and controls and odds ratio estimates for colorectal cancer

SNP	Chromosome	Gene	Allele(A/B)	HWE-*P*^a^	MAF-case	MAF-control	OR(95%CI)	*P*^b^
rs10069690	5	TERT	T/C	0.347	87(17.9%)	85(14.36%)	1.30(0.94-1.8)	0.114
rs2242652	5	TERT	A/G	0.523	88(17.81%)	96(16%)	1.14(0.83-1.56)	0.425
rs2853677	5	TERT	G/A	0.696	183(37.04%)	199(33.17%)	1.19(0.92-1.52)	0.181
rs2853676	5	TERT	T/C	0.817	90(18.22%)	88(14.67%)	1.3(0.94-1.79)	0.113
rs3792792	5	TNIP1	C/T	1	33(6.68%)	37(6.17%)	1.09(0.67-1.77)	0.729
rs7708392	5	TNIP1	G/C	0.861	141(28.54%)	125(20.83%)	1.52(1.15-2)	0.003*
rs10036748	5	TNIP1	C/T	0.861	141(28.54%)	125(20.83%)	1.52(1.15-2)	0.003*
rs3814220	10	OBFC1	G/A	0.067	146(29.55%)	197(32.83%)	0.86(0.66-1.11)	0.245
rs12765878	10	OBFC1	C/T	0.067	147(29.76%)	197(32.83%)	0.87(0.67-1.12)	0.275
rs11191865	10	OBFC1	A/G	0.067	148(29.96%)	197(32.83%)	0.88(0.68-1.13)	0.309
rs9420907	10	OBFC1	C/A	1	7(1.42%)	4(0.67%)	2.14(0.62-7.36)	0.351^†^

SNPs: single nucleotide polymorphisms; A: miner allele B: major allele; MAF: minor allele frequency; HWE: Hardy-Weinberg equilibrium; OR: odds ratio; CI: confidence interval. *P* ^a^, Hardy-Weinberg equilibrium (HWE) *P* value was calculated by exact test. *P* ^b^ value was calculated by Pearson's chi-square test. † indicated the *P* value calculated by Continuity Correction test.*: *P*<0.05 indicates statistical significance.

We further assessed the association between prominent polymorphism loci and CRC risk under four models (genotype, dominant, recessive, and additive model) using unconditional logistic regression analysis adjustment for gender and age in Table 3. The results showed that rs10069690 had a modest association with CRC risk in genotype model (C/T *vs* C/C OR=1.49, 95%CI=1-2.22, *P*=0.049). And an extremely significant association was found between rs7708392 and CRC risk in genotype model (C/G *vs* C/C OR=1.86, 95%CI=1.3-2.66, *P*=0.0007), dominant model (C/G-G/G *vs* C/C OR=1.82 95%CI=1.28-2.58, *P*=0.0008), and additive model (OR=1.58, 95%CI=1.17-2.13, *P*=0.003). At the same time, rs10036748 was also associated with increased the CRC risk in genotype model (T/C *vs* T/T OR=1.86, 95%CI=1.3-2.66, *P*=0.0007), dominant model (T/C-C/C *vs* T/T OR=1.82, 95%CI=1.28-2.58, *P*=0.0008) and additive model (OR=1.58 95%CI=1.17-2.13, *P*=0.003). In addition, rs12765878 and rs11191865 might be a protective role in CRC risk under genotype model (T/C *vs* T/T OR=0.69, 95%CI=0.48-1, *P*=0.048; G/A *vs* G/G OR=0.68, 95%CI=0.48-0.98, *P*=0.041 respectively). After Bonferroni's multiple adjustment applied to our date, we were pleasantly surprised to find that rs7708392 and rs10036748 were still significantly associated with CRC risk. The associations of other SNPs were disappeared.

**Table 3:** Genotype distributions of prominent SNPs under model and their association with the risk of developing colorectal cancer

SNP	Model	Genotype	Case(%)	Control(%)	Adjustment analysis	
					OR(95%CI)	*P*
rs10069690	Genotype	C/C	163(67.08%)	219(73.99%)	1	
		C/T	73(30.04%)	69(23.31%)	1.49(1-2.22)	0.049*
		T/T	7(2.88%)	8(2.7%)	1.11(0.39-3.22)	0.843
	Dominant	C/C	163(67.08%)	219(73.99%)	1	
		T/C-T/T	80(32.92%)	77(26.01%)	1.45(0.99-2.13)	0.057
	Recessive	C/C-C/T	236(97.12%)	288(97.3%)	1	
		T/T	7(2.88%)	8(2.7%)	1(0.35-2.87)	1
	Additive	-	-	-	1.32(0.95-1.85)	0.099
rs7708392	Genotype	C/C	117(47.37%)	187(62.33%)	1	
		C/G	119(48.18%)	101(33.67%)	1.86(1.3-2.66)	0.0007*
		G/G	11(4.45%)	12(4%)	1.47(0.62-3.5)	0.384
	Dominant	C/C	117(47.37%)	187(62.33%)	1	
		C/G-G/G	130(52.63%)	113(37.67%)	1.82(1.28-2.58)	0.0008*
	Recessive	C/C-C/G	236(95.55%)	288(96%)	1	
		G/G	11(4.45%)	12(4%)	1.13(0.48-2.66)	0.774
	Additive	-	-	-	1.58(1.17-2.13)	0.003*
rs10036748	Genotype	T/T	117(47.37%)	187(62.33%)	1	
		T/C	119(48.18%)	101(33.67%)	1.86(1.3-2.66)	0.0007*
		C/C	11(4.45%)	12(4%)	1.47(0.62-3.5)	0.384
	Dominant	T/T	117(47.37%)	187(62.33%)	1	
		T/C-C/C	130(52.63%)	113(37.67%)	1.82(1.28-2.58)	0.0008*
	Recessive	T/T-T/C	236(95.55%)	288(96%)	1	
		C/C	11(4.45%)	12(4%)	1.13(0.48-2.66)	0.774
	Additive	-	-	-	1.58(1.17-2.13)	0.003*
rs12765878	Genotype	T/T	125(50.61%)	128(42.67%)	1	
		T/C	97(39.27%)	147(49%)	0.69(0.48-1)	0.048*
		C/C	25(10.12%)	25(8.33%)	0.98(0.53-1.83)	0.957
	Dominant	T/T	125(50.61%)	128(42.67%)	1	
		T/C-T/T	122(49.39%)	172(57.33%)	0.74(0.52-1.04)	0.082
	Recessive	T/T-T/C	222(89.88%)	275(91.67%)	1	
		C/C	25(10.12%)	25(8.33%)	1.18(0.65-2.14)	0.596
	Additive	-	-	-	0.86(0.66-1.12)	0.268
rs11191865	Genotype	G/G	125(50.61%)	128(42.67%)	1	
		G/A	96(38.87%)	147(49%)	0.68(0.48-0.98)	0.041*
		A/A	26(10.53%)	25(8.33%)	1.04(0.56-1.92)	0.907
	Dominant	G/G	125(50.61%)	128(42.67%)	1	
		G/A-A/A	122(49.39%)	172(57.33%)	0.74(0.52-1.04)	0.082
	Recessive	G/G-G/A	221(89.47%)	275(91.67%)	1	
		A/A	26(10.53%)	25(8.33%)	1.25(0.69-2.26)	0.463
	Additive	-	-	-	0.87(0.67-1.14)	0.313

SNPs: single-nucleotide polymorphisms; OR: odds ratio; CI: confidence interval. *P* ^a^ values were calculated by Wald’ test with adjustment for gender and age. *P<0.05 indicates statistical significance. Bonferroni's multiple adjustment was applied to the level of significance, which was set at *P* < 0.0011 (0.05/44).

### Haplotype analysis

**Figure 1:**
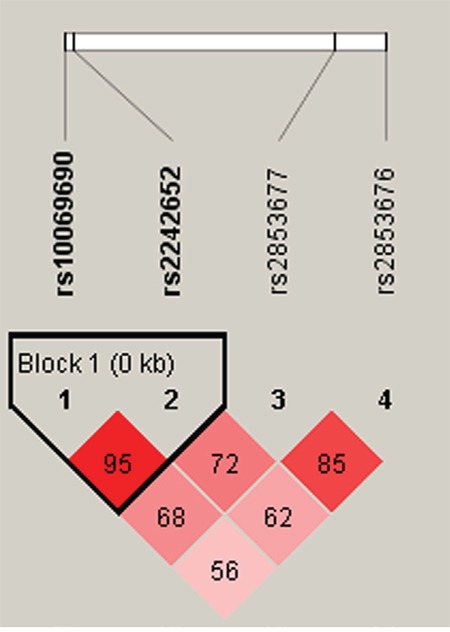
Haplotype block map for part of the SNPs in the *TERT* gene There were there SNPs in the haplotype map, and linkage disequilibrium block containing two SNPs: rs10069690-rs2242652. Standard color frame is used to show LD pattern. The D value was 0.95. These two SNPs tended to be co-inherited.

**Figure 2:**
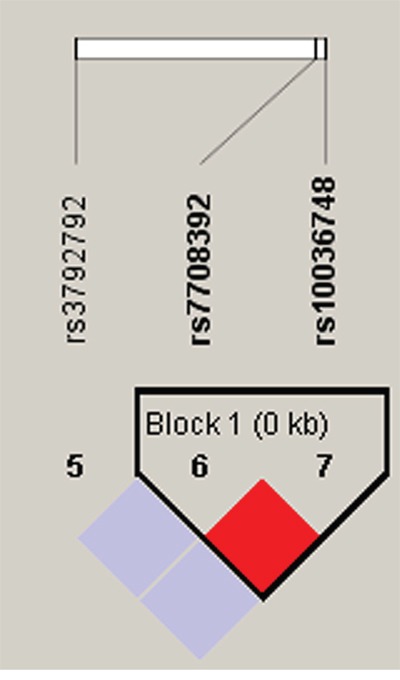
Haplotype block map for part of the SNPs in the *TNIP1* gene One block in the figure showed higher LD, and the linkage disequilibrium block was composed of two SNPs rs7708392 and rs10039748. That the D value was 1 indicated strong linkage disequilibrium between the two SNPs.

**Figure 3:**
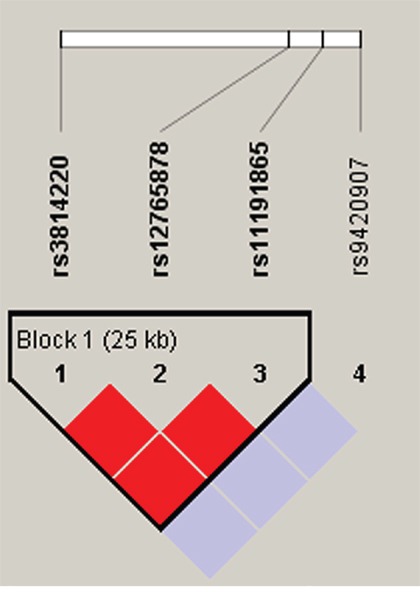
Haplotype block map for part of the SNPs in the *OBFC1* gene Darker shades of red indicate higher D’ and display statistically significant associations between a pair of SNPs. Rs3814220-rs12765878-rs11191865 block in the figure was found have the linkage disequilibrium, and the D value was 1.

Lastly, we performed haplotype analysis for the *TERT*, *TNIP1* and *OBFC1* genes and found 3 strong linkage of these candidate SNPs, containing rs10069690-rs2242652 block in *TERT* (Figure 1), rs7708392-rs10039748 block in *TNIP1* (Figure 2), and rs3814220-rs12765878-rs11191865 block in *OBFC1* (Figure 3). The associations between haplotypes of the 3 blocks and the CRC risk are listed in Table 4. We found that “C-A” haplotype frequency of rs10069690-rs2242652 block was statistically significant difference between cases and controls (0.002 *vs* 0.017, *P*=0.016), but this haplotype was not significantly reduced the CRC risk (OR=0.14, 95%CI=0.02-1.15, *P*=0.068). Furthermore, two haplotypes of rs7708392-rs10039748 block were found extremely significant associated with CRC risk, “G-C” haplotype with an increased the CRC risk (OR=1.58, 95%CI=1.17-2.13, *P*=0.003), the other “C-T” haplotype with a decreased the CRC risk (OR=0.63, 95%CI=0.47-0.86, *P*=0.003). No significant association was found in rs3814220-rs12765878-rs11191865 block.

**Table 4:** Haplotype frequencies and the association with colorectal cancer risk in case and control subjects

Gene	SNPs	Haplotype	Freq (case)	Freq (control)	*P*^a^	OR (95% CI)	*P*^b^
TERT	rs10069690|rs2242652	T-A	0.171	0.139	0.143	1.29(0.92-1.81)	0.133
		C-A	0.002	0.017	0.016*	0.14(0.02-1.15)	0.068
		C-G	0.819	0.84	0.37	0.84(0.6-1.16)	0.286
TNIP1	rs7708392|rs10036748	G-C	0.285	0.208	0.003*	1.58(1.17-2.13)	0.003*
		C-T	0.715	0.792	0.003*	0.63(0.47-0.86)	0.003*
OBFC1	rs3814220|rs12765878|rs11191865	G-C-A	0.296	0.328	0.245	0.85(0.65-1.11)	0.239
		A-T-G	0.7	0.672	0.309	1.15(0.88-1.5)	0.313

*P*^a^ values were calculated by Pearson's chi-square test; *P*^b^ values were calculated by Wald' test adjustment for gender and age;

*: *P* < 0.05 indicates statistical significance.

## DISCUSSION AND CONCLUSION

The present research examined 11 allelic variants within the telomere length-related *TERT*, *TNIP1* and *OBFC1* genes and their implication on CRC risk in Chinese Han population. Overall, the results demonstrated that polymorphisms of rs7708392 and rs10039748 in *TNIP1* had a significant influence on CRC susceptibility for the first time. Moreover, these two SNPs exhibited increase the CRC risk in genotype model, dominant model and additive model after Bonferroni's correction. Under haplotype analysis, we not only found that the haplotype “G/C” of *TNIP1* was associated with a 1.58-fold enhancive the CRC risk, but also found haplotype “CT” significantly decreased the CRC risk by 0.63-fold. These finds indicated that the polymorphism of *TNIP1* gene was significantly correlated with the risk of CRC in Chinese Han population, and *TNIP1* may play a key role in CRC progression through aberrantly activate the NF-κβ-induced signaling.

*TNIP1*, located in 5p33.1, encodes an A20-binding protein which can regulate the activation of nuclear factor kappa-β (NF-κβ) [14]. NF-κβ is a transcriptional factor often implicated in a variety of different diseases [15], and article has indicated a critical link between cancers and NF-κβ which plays a crucial role in oncogenesis and the metastasis of tumors through a variety of different ways [16]. Furthermore evidence revealed that NF-κβ is constitutively activated in human colorectal carcinoma tissue [17] and play a key role in colorectal carcinoma progression through activation of its downstream target genes [18]. For example, the decreased expression of NF-κβ could inhibit cell cycle progression in CRC cell lines, and arrest in the G0/G1 phase through down-regulation of its target genes E2F1 and TS [19]. In addition, both reactive oxygen species and inflammation reaction also contribute to the connection between NF-κβ and CRC [20, 21]. Some studies have suggested that the polymorphism of NF-κβ1 and NF-κβ appeared to jointly contribute to CRC risk in the southern Chinese population [22], and the polymorphism of NF-κβ was related to susceptibility and prognosis of CRC patients [23].

However, studies about the association between *TNIP1* and colorectal cancer are rarely. Interestingly, many researchers are interested in finding the polymorphism of *TNIP1* associated with systemic lupus erythematosus (SLE). Research reported that the risk allele “G” of rs7708392 in *TNIP1* has a higher frequency in Asian than in Caucasian, and rs7708392 is a shared SLE susceptibility SNP in the Asian and Caucasian populations [24, 25]. Similarly, rs10039748 within intron 1 of *TNIP*1 also showed significant association with the susceptibility of SLE, systemic sclerosis [26] and asthma [27]. In this study, a novel association between *TNIP1* polymorphisms and CRC risk was found, and this link might emerge through the effect of *TNIP1* gene on transcriptional regulation of NF-κβ. We hypothesized that the variant of rs7708392 and rs10039748 might alter the function of A20-binding protein, and thus affect the NF-κβ-induced signal pathway to increase the tumor promotion and maintenance. In the future, the mechanism of *TNIP1* polymorphisms in the pathogenesis of CRC need to be further elucidated.

*TERT* gene is mapped on chromosome 5p15.33, a genomic region which was confirmed to be associated with multiple cancer types. In this current study, we studied the effect of four SNPs from *TERT* gene on CRC risk in Chinese Han population and found that none of the investigated SNPs were associated with CRC risk. Similar to our results, the risk allele “T” of rs2853676 has been identified not associated with CRC risk in a previous involving an Austrian population [28]. A meta-analysis, rs2853676 in *TERT* have been identified as being associated with glioma, lung and ovarian cancer, but not melanoma, breast, pancreatic and colorectal cancer [29]. In addition, rs10069690, rs2242652 and rs2853676 were also confirmed not associated with colon and rectal cancer, however, after interacted with body mass index, rs10069690 and rs2242652 showed a significant association with risk of colon cancer in the California population [30]. Our data also did not confirm the correlation between the variant of these four SNPs in *TERT* and CRC risk in Chinese Han population.

Except the two genes *TNIP1* and *TERT*, we also selected four SNPs from *OBFC1* gene, which is located in 10q24.33. Polymorphisms of rs3814220, rs12765878 and rs9420907 have been identified associate with leukocyte telomere length in a meta-analysis [7]. In addition, susceptibility loci rs11191865 was a share risk loci for idiopathic interstitial pneumonia and systemic sclerosis [31]. However, in this case control study, the four SNPs in *OBFC1* don't have a correlation with CRC risk in Chinese Han population. Despite the current study possessing the energy, the negative results of major SNPs in this study may convert into positive ones when the sample size of CRC samples is large enough.

In conclusion, our present study firstly provides evidence that rs7708392 and rs10039748 in the *TNIP1* gene are associated with the risk of CRC in Chinese Han population. It is possible that *TNIP1* might be a new precursory biomarker to CRC and increase the possibility of developing CRC. Our result can provide a theoretical foundation for other researchers to further study the correlation between *TNIP1* and susceptibility to CRC in Chinese Han population or other populations.

## MATERIALS AND METHODS

### Study subjects

We evaluate the influence of the thirteen variants on susceptibility to colorectal cancer with 247 cases and 300 controls in Chinese Northwest Population. All the cases were treated at the First Affiliated Hospital of Xi’an Jiaotong University between June 2013 and May 2016. According to the guidance statement described by Qaseem A et al [32], the diagnosis criteria of CRC contain clinical history, routine laboratory evaluation and histopathological detection, and all the cases were patients with positive colonoscopic results for malignancy. The inclusion criteria of the cases also contained the expected survival of more than three months, and qualified hematology, liver and kidney function. In addition, the controls included individuals undergoing colonoscopy for various gastrointestinal complaints and sampled at the same time as the cases. The controls were negative colonoscopic results for malignancy and never had any type of disease, including cardiovascular diseases, hepatic disease and pulmonary diseases. All subjects were interviewed by a nurse who collected detailed information about gender, age, region and ethnicity.

### Ethics committee statement

The use of human tissue and the protocol in this study were strictly conformed in accordance with the tenets of the Declaration of Helsinki and were approved by the Ethical the First Affiliated Hospital of Xi’an Jiaotong University and Xi’an Jiaotong University for approval of research involving human subjects. The individual in this manuscript has given written informed consent to publish these case details.

### SNP selection and genotyping

In this case-control study, we selected 4 SNPs in *TERT* (rs10069690, rs2242652, rs2853677, rs2853676), 3 SNPs in *TNIP1* (rs3792792, rs7708392, rs10036748) and 4 SNPs in *OBFC1* (rs3814220, rs12765878, rs11191865, rs9420907), each with a minor allele frequency (MAF) higher than 5% in the Han Chinese population. Genomic DNA extracted from whole blood by GoldMag extraction method (GoldMag Co. Ltd, Xi’an, China) [33], and then DNA was stored at −80°C. Spectrometry (DU530UV/VIS spectrophotometer, Beckman Instruments, Fullerton, CA, USA) was used to measure the DNA concentration. According to the manufacturer's protocols, Sequenom MassARRAY® RS1000 system (Agena Bioscience Inc., San Diego, CA, USA) was used to perform genotyping, and the Sequence MassARRAY Assay Design 4.0 (Agena Bioscience Inc.) software was used to design a Multiplexed SNP mass-EXTEND assay [34]. After genotyping, Sequenom Typer 4.0 Software (Agena Bioscience Inc.) was used to conduct data analysis [35].

### Statistical analysis

SPSS 17.0 statistical packages (SPSS, Chicago, IL) were used to perform the differences of subjects’ characteristic containing gender and age. All *P* values were two-sided, and values of *P*≤0.05 were considered significant. The genotype frequency distribution of each SNP needs accord with Hardy-Weinberg Equilibrium (HWE) in controls, and exact test was used to check the feature. The alleles and genotype frequencies among cases and controls were calculated by Chi-squared test or Continuity Correction test. Odds ratios (OR) and 95% Confidence Intervals (CI) were used to evaluate the association between genotypes and CRC risk in four models (genotype, dominant, recessive and additive model) using unconditional logistic analysis adjusted for age and gender. In order to eliminate the probability of false-positive results, Bonferroni's multiple adjustment was applied to the level of significance, which was set at *P* < 0.0011 (0.05/44). Finally, SHEsis software platform [36] and Haploview software package (version 4.2) (Broad Institute, Cambridge, MA, USA) was used to perform the linkage disequilibrium (LD), and to analysis the association at polymorphic loci [37]. The D ≥ 0.8 indicated that a pair of SNPs had a significant LD, and tended to co-inheritance.

## Abbreviations

CRC: colorectal cancer
SNP: single nucleotide polymorphism
OR: odds ratio
CI: confidence interval
LD: linkage disequilibrium
